# No consistent effect of daytime versus night-time measurement of thermal tolerance in nocturnal and diurnal lizards

**DOI:** 10.1093/conphys/coac020

**Published:** 2022-04-20

**Authors:** Pauline C Dufour, Toby P N Tsang, Susana Clusella-Trullas, Timothy C Bonebrake

**Affiliations:** Area of Ecology & Biodiversity, School of Biological Sciences, Kadoorie Biological Sciences Building, The University of Hong Kong, Pok Fu Lam Road, Hong Kong Special Administrative Region, China; Area of Ecology & Biodiversity, School of Biological Sciences, Kadoorie Biological Sciences Building, The University of Hong Kong, Pok Fu Lam Road, Hong Kong Special Administrative Region, China; Department of Botany and Zoology, Stellenbosch University, Private Bag X1, Stellenbosch 7602, South Africa; Area of Ecology & Biodiversity, School of Biological Sciences, Kadoorie Biological Sciences Building, The University of Hong Kong, Pok Fu Lam Road, Hong Kong Special Administrative Region, China

## Abstract

While essential in understanding impacts of climate change for organisms, diel variation remains an understudied component of temporal variation in thermal tolerance limits [i.e. the critical thermal minimum (CTmin) and maximum (CTmax)]. For example, a higher Ctmax might be expected for an individual if the measurement is taken during the day (when heat stress is most likely to occur) instead of at night. We measured thermal tolerance (Ctmin and Ctmax) during both the daytime and night-time in 101 nocturnal and diurnal geckos and skinks in Hong Kong and in South Africa, representing six species and covering a range of habitats. We found that period of measurement (day vs. night) only affected Ctmin in South Africa (but not in Hong Kong) and that Ctmax was unaffected. Body size and species were important factors for determining Ctmax in Hong Kong and Ctmin in South Africa, respectively. Overall, however, we did not find consistent diel variation of thermal tolerance and suggest that measurements of critical thermal limits may be influenced by timing of measurement—but that such effects, when present, are likely to be context-dependent.

## Teaser

Thermal tolerance is a critical metric for understanding climate change impacts on biodiversity—but diel variation remains understudied. Across several lizard species in Hong Kong and South Africa, we found no consistent effect of measurement timing (day or night) on thermal tolerance, but one species exhibited a higher daytime critical thermal minimum.

## Introduction

Life on Earth relies on temperature being within particular limits and a large part of this temperature variation depends on the availability of solar radiation. Temperature varies between day and night, and the amplitude of this change can vary greatly across space (e.g. temperate, tropical or polar regions) and time (seasons) (Ghalambor *et al.*, 2006; Janzen, 1967). Because of their reliance on external sources to achieve desired optimal temperatures, lizards and ectotherms in general are good candidates to study the effects of such variation (Clusella-Trullas and Chown, 2014). They can buffer diel temperature variation with behavioural and physiological thermoregulation, depending on diurnal and nocturnal patterns.

The parameters of thermal performance curves, such as optimal temperature and thermal tolerance range, can vary depending on the amplitude of diel temperature variation (Bonino *et al.*, 2015), the capacity to buffer climatic variation among species (Addo-Bediako *et al.*, 2000; Clusella-Trullas *et al.*, 2011; Grigg and Buckley, 2013; Sunday *et al.*, 2014) and the region studied (e.g. tropical vs. temperate; Deutsch *et al.*, 2008; Sunday *et al.*, 2011). The climatic variability hypothesis (CVH) states that a more thermally variable environment should lead to a wider thermal tolerance range, while thermal stability should lead to more thermal specialists (Addo-Bediako *et al.*, 2000; Baudier *et al.*, 2018; Gaston, 2009; Ghalambor *et al.*, 2006; Janzen, 1967). Therefore, thermal tolerance ranges are likely to vary as a function of diel thermal fluctuation (Clusella-Trullas and Chown, 2014). The effects of thermal variation on physiological responses or development are also species-dependent (Kern *et al.*, 2015). Thermal niches in the context of diel variation for ectotherms must also consider the influence of active and inactive phases for population growth and species performance (Gvoždík, 2018). For example, during summer, night-time is often a key recovery period after daytime extreme heat exposure (Bai *et al.*, 2019), and climate change is causing a general increase in the number of warm nights (Davy *et al.*, 2017; Stocker *et al.*, 2013).

Within the context of CVH, diel variation of thermal tolerance could have implications in drawing conclusions for climate change impacts on ectotherms. We define critical thermal limits (CTLs) as the temperature beyond which ectotherms cannot function or respond in a coordinated manner (Clusella-Trullas *et al.*, 2011; Ward *et al.*, 1993). The lower CTL is referred to as the ‘Critical Thermal Minimum’ (Ctmin) and the higher CTL as ‘Critical Thermal Maximum’ (Ctmax). To date, the main compilation of CTLs across the available literature (Bennett *et al.*, 2018) prioritized data measured during active phases. However, despite the large presence of thermal tolerance in the thermal physiology literature (Bennett *et al.*, 2021), little research has been carried out on the existence of diel variation in CTLs, especially for lizards (Clusella-Trullas and Chown, 2014). In the Australian agamid Jacky lizard, the lowest panting thresholds—the Tb at which animals begin to rapidly breathe with their mouths gaping to increase evaporative cooling (Taylor *et al.*, 2021)—were observed at night, during the resting phase of this diurnal species (Chong *et al.*, 1973). Other examples from other taxa largely focus on single species in specific locations. For example, daily rhythms of Ctmax were observed in tadpoles of *Rana clamitans*, with the lowest tolerance achieved during the night and being highest around noon (Willhite and Cupp Jr, 1982). Other amphibians, such as *Bufo marinus* also display diel patterns in heat resistance, with the onset of spasms (as a measure of Ctmax) being triggered at lower temperatures during the night than during the day, but also exhibiting more consistency during the day (Johnson, 1972). In insects, Ribeiro *et al.* (2012) found no diel variation in Ctmax for an ant species in Brazil (*Atta laevigata*) and Bai *et al.* (2019) found that the Ctmax of lady beetle larvae was lower when measured in the morning following night-time recovery relative to the evening.

Over the past decade, stress physiology has become an important component of research in understanding the successes or failures of conservation practices, for example during reintroductions (Turko *et al.*, 2021), translocations (Cooke *et al.*, 2001) or management of fish stocks (Cooke *et al.*, 2012). Thermal physiology is also an important factor to determining the success of invasive species (Zerebecki and Sorte, 2011) or monitoring outcomes of conservation measures (Madliger *et al.*, 2018). Integrating physiology, in particular thermal ecology, into conservation decisions has aided in the management of translocated individuals of endangered species (Besson and Cree, 2011). These major breakthroughs are only possible through an understanding of thermal requirements, including thermal limits at different life stages, reproductive status (Virens and Cree, 2019) and metabolism (Besson and Cree, 2011). Diel variation is another important source of thermal variation that is generally overlooked. If daily variation in thermal tolerance follows daily variation in air temperatures, having a lower tolerance for cold or heat at certain parts of the day could result in dramatic consequences for habitat suitability of ectotherms and their reproductive success (Cree *et al.*, 2020).

In this study, we examined diel variation of thermal tolerance limits (both Ctmin and Ctmax) using multiple lizard species found in two different climatic regions (Hong Kong and South Africa). We asked whether diel variation in temperature influenced diel variation in thermal tolerance for lizards. Lizards are widely distributed across latitude, inhabit various habitats and climates, include nocturnal, diurnal, crepuscular and cathemeral species, and their thermal biology has generated an extensive literature (Angilletta, 2009; Grigg and Buckley, 2013; Huey and Kingsolver, 1989). Additionally, as climate change has been highlighted as a major threat to lizards worldwide, directly and indirectly (Buckley *et al.*, 2015; Kubisch *et al.*, 2016; Pontes-da-Silva *et al.*, 2018; Sinervo *et al.*, 2010), it is essential to understand their sensitivity to temperature and rely on accurate values to forecast their vulnerability to elevated temperatures. We hypothesized that Ctmax would be higher during the day when temperatures are highest, while at night (when thermal stresses are unlikely to be significant), Ctmax would be lower. Conversely, we hypothesized that Ctmin would be lowest during the night relative to the day.

## Materials and methods

### Study species and sites

We chose common species representing a diversity of activity patterns, including diurnal, nocturnal and cathemeral (i.e. active during the day and/or night) species. Our objective was to cover a large diversity in terms of climates and temporal niches (i.e. activity periods). We collected lizards from two different countries experiencing different climates: Hong Kong SAR, China and the Western Cape province of South Africa. In Hong Kong (Supplementary Material Fig. S1), our study species were two common and widespread nocturnal geckos (Chinese gecko *Gekko chinensis* and Bowring’s gecko *Hemidactylus bowringii*), as well as the rarer cathemeral Chinese waterside skink (*Tropidophorus sinicus*). In South Africa (Supplementary Material Fig. S2), we chose to focus on three common lizard species: one nocturnal gecko (Bibron’s thick-toed gecko *Chondrodactylus bibronii*), one diurnal gecko (Cape dwarf gecko *Lygodactylus capensis*) and one diurnal skink (Red-sided skink *Trachylepis homalocephala*).

The humid and monsoon-influenced sub-tropical climate (Köppen climate classification Cwa) of Hong Kong is characterized by tropical summers and more temperate-like winters. Summers are long, with high relative humidity (in the 80–90% RH range) and account for ~80% of the annual rainfall (data from Hong Kong Observatory, https://www.hko.gov.hk/). Winters are generally mild (mean temperatures of the three coldest months: 17.27°C; data from Hong Kong Observatory, https://www.hko.gov.hk/), with a short (~2 weeks) drop in temperature during the coldest month (usually January), reaching close-to-zero degrees Celsius in mountainous areas. The Western Cape is about 450 times the size of Hong Kong and offers a wide range of terrains and climates. Our sampled areas ranged from hot- and warm-Mediterranean climates (hot and dry summer, cold and wet winter; Köppen–Geiger climate classification Csa and Csb) in the South, to cold semi-arid regions in the North and East (hotter and drier summers than in the South, cold dry winters, large daily fluctuations in temperature; Köppen climate classification Bsk; Adesina *et al.*, 2016; Beck *et al.*, 2018). In Hong Kong, we sampled geckos and skinks between April and August 2019, i.e. the warmest months with the highest levels of activity for lizards, in three different locations on Hong Kong Island and Lamma Island (Supplementary Material Fig. S1). Habitats of Hong Kong geckos are mostly composed of secondary forest patches, in the surroundings of or within villages. In South Africa, sampling took place in five different sites in the Western Cape (Supplementary Material Fig. S2) during the austral springs of 2018 and 2019 (September–November): one site for *L. capensis* (Csa climate; https://en.climate-data.org/), one site for *T. homalocephala* (Csb climate) and three sites for *C. bibronii* (Bsk climate). Lizards were found on rock outcrops (*C. bibronii* and *T. homalephala*) and on exotic trees next to a plant nursery (*L. capensis*). Exact locations are not provided as to prevent illegal collection of wild animals (following recommendations from Lindenmayer and Scheele, 2017), but rough locations can be seen on Supplementary Material Fig. S2. Moreover, we retrieved microclimatic data from Kearney *et al.* (2014) for all five sites in South Africa and one site (out of three) for Hong Kong. The two missing HK sites are not available by the microclim model as they are on a small island, but climate between these three sites is similar (https://www.hko.gov.hk/en/cis/). We downloaded air temperature at 120 cm from the ground and 50% of shade (thereafter ‘ambient’) with rock substrate temperatures, i.e. the substrate most relevant to our study species, at three levels of shade cover i.e. 0%, 50% and 100% (Supplementary Material Fig. S3).

### Thermal tolerance limit assays

We here define critical minimum temperature (Ctmin) and critical maximum temperature (Ctmax) of lizards as the temperature at which they lose their locomotory function (Hutchison, 1961; Jacobson and Whitford, 1970; Lowe and Vance, 1955; Muñoz *et al.*, 2014; Spellerberg, 1973). Animals were brought back from the field to university laboratories (The University of Hong Kong or Stellenbosch University) and were kept for 7 days prior to the experiments (Allen *et al.*, 2016; Weldon *et al.*, 2011) at room temperature (23–24°C in Hong Kong, 24–26°C in South Africa). They were maintained in individual plastic terraria with substrate and shelter with a 12D:12N light cycle and the ability to thermoregulate under a heating bulb (reaching up to 30°C on one side of their terrarium between 10 am and 4 pm and room temperature at the other end). Actual sunrise and sunset times were ~6 am and ~6:30 pm in Hong Kong and ~5:30 am and 7–7:30 pm in South Africa, respectively. Lizards were fed every other day with two crickets and/or mealworms. Water was provided daily.

We placed each individual in a small metal container that was then emerged into a programmable water bath (R4-GP200 in South Africa, R4-TX150 in Hong Kong, Grant Instruments, Cambridge, UK) to keep the temperature uniform within the container. The container was covered by a plastic sheet pierced by holes and sealed by adhesive paste. This way, the animal could breathe and move but could not escape. The programme started with a 12-minute equilibration period at 25°C followed by a heating (Ctmax) or cooling (Ctmin) phase during which temperature was either increased (Ctmax) or decreased (Ctmin) at a constant rate of 0.5°C/min (Allen *et al.*, 2016). Every degree after 35°C (Ctmax), or below 10°C (Ctmin), we checked for the ‘loss of righting response’, i.e. when placed on the back and stimulated, the lizard was not able to right itself (see Brattstrom, 1970; Leal and Gunderson, 2012). We monitored the container temperature at all times by inserting thermocouples inside the container—either at the bottom of the container (skinks) or against the side of the container (geckos) depending on where the animals usually spent the duration of the experiment—and only measured the cloacal temperature of animals at the end. Temperature of the container and of the cloaca were highly correlated (Hong Kong: *R^2^* = 0.99*, P* < 0.001; South Africa: R^2^ = 0.98, *P* < 0.001), but since handling time of the animal at the end varied, we chose to focus on container temperature as a proxy for body temperature. Immediately after they reached the CTL, the lizard was removed from its container and was placed at room temperature and in a moist environment until full recovery.

**Figure 1:**
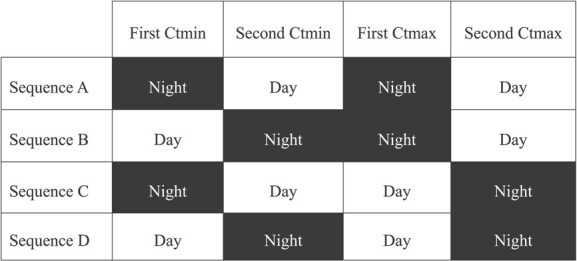
Experimental design: each lizard went through one of the four sequences of experiments (A, B, C or D) with two Ctmin (day or night measurement) followed by two Ctmax (day or night measurement).

Each lizard was subjected to four experimental treatments: two Ctmin (night first then day, or day first then night) followed by two Ctmax (night first then day, or day first then night). We chose to run our experiments between 10 am and 4 pm for ‘day’, and between 8 pm and 11 pm for ‘night’, when animals were observed to be fully active in the wild for diurnal and nocturnal species, respectively. There were always 48 hours between two experiments to allow for the animal to recover fully. We chose to always begin with Ctmin as to minimize the risks associated with lethal effects caused by exposure to high temperatures. Each individual followed a semi-randomized sequence of experiments, always starting by two Ctmin followed by two Ctmax, with the period of measurement (i.e. either day or night), varying so all possible combinations were represented to limit experimental sequence effects (Fig. 1).

### Data analyses

Each lizard underwent two Ctmin and two Ctmax measurements during its captivity starting at different hours. We tested for the potential effect of the sequence (see Fig. 1) and trial number on either Ctmin or Ctmax using a two-way ANOVA with sequence (A, B, C or D) and trial number (first or second Ctmin, first or second Ctmax) as the explanatory variable.

This present work is principally designed to understand the effect of period (night or day) on Ctmin and Ctmax, but we also tested for species, sex and snout-vent length (SVL), as well as the interaction between period and those variables. To do so, we started with models using the *lme4* package in R (Bates *et al.*, 2007), with Ctmin or Ctmax as the response variables, and period, species, sex, SVL and the first order interaction between period and the other variables, as fixed effects. We also included two random effects: (i) hour of day (rounded to the closest hour) as the time we started the experiment and (ii) individual. The random effect structure was fixed in the model selection process, while interaction terms that omit period or higher than first order interactions were constrained from selection. We used the *dredge()* function, from the *MuMIn* package (Bartón, 2020), to perform an automated model selection through all possible models. The best fitting models were selected when ΔAIC_c_ < 2 (Burnham and Anderson, 2004) and averaged with the *model.avg()* function. We chose to report the full average of all models, a more conservative approach than the use of the conditional average, as this approach obtains coefficient averages across models by assuming an estimate of zero in models for which the parameter was not included (Grueber *et al.*, 2011). Models were also separated by region, i.e. we performed two separate analyses for Hong Kong and for South Africa. In South Africa, lizard species were strikingly different in size (Table 1), such that species and size were highly correlated and therefore only species was included in those analyses. We also included trial number for Ctmin in South Africa as a follow-up to our preliminary analysis. We checked for linearity of the residuals of the best-fitted model and no data transformation was deemed necessary. We also performed the same model selection using Thermal Tolerance Breadth (TTB), which is the difference between Ctmax and Ctmin, as the response variable.

**Table 1:** Characteristics of species represented in our study: provenance, activity period, SVL (mm), body mass (g) and sample size

Location	Species	Activity period	SVL (mm)^a^	Body mass (g)^a^	*n*
Hong Kong	*G. chinensis*	Nocturnal	61.1 ± 7.14	4.93 ± 1.49	30
	*H. bowringii*	Nocturnal	46.85 ± 3.92	2.34 ± 0.62	19
	*T. sinicus*	Cathemeral	59.01 ± 7.01	5.09 ± 1.98	4
South Africa	*C. bibronii*	Nocturnal	75.54 ± 8.62	19.27 ± 14.75	26
	*L. capensis*	Diurnal	32.95 ± 1.44	0.87 ± 0.21	12
	*T. homalocephala*	Diurnal	57.73 ± 6.56	4.72 ± 1.74	9

^a^Calculated as mean ± standard deviation.

## Results

We captured a total of 54 lizards (50 geckos and 4 skinks) in Hong Kong and 47 lizards (38 geckos and 9 skinks) in South Africa (Table 1). The order in which we performed the experiments (i.e. sequence A, B, C or D) and the trial number (1, 2, 3 or 4) did not affect Ctmin nor Ctmax in Hong Kong (*P = 0.64* and *P = 0.66*, respectively) nor Ctmax in South Africa (*P* = 0.34). However, in South Africa we found a small effect of the sequence on Ctmin, with the second Ctmin resulting on average in lower values than the first Ctmin A by 0.8°C (two-way ANOVA, *R^2^ = 0.05, P = 0.046*). Trial number was therefore included in further analyses of Ctmin in South Africa.

We averaged two to five best models for each region and thermal limit analyses (Table 2). Each model included period (night or day) and sex as fixed effects. Two averaged models (Ctmax in Hong Kong and Ctmin in South Africa) also included interaction terms between SVL and period (Ctmax in Hong Kong) and species and period (Ctmax in Hong Kong and Ctmin in South Africa). SVL was selected in the best models in Hong Kong (Table 2).

**Table 2:** Candidate models explaining Ctmax and Ctmin in Hong Kong and South Africa

Response variable	Model	Fixed effects	Random effects	Number of best models in the average
Ctmax (HK)	Model 1	period + SVL + sex + period*sex + period^*^SVL	Hour of day + individual	4
Ctmax (SA)	Model 2	period + sex	Hour of day + individual	2
Ctmin (HK)	Model 3	period + species + sex + SVL	Hour of day + individual	5
Ctmin (SA)	Model 4	Period + sex + species + period * species + period ^*^ sex + Trial number	Hour of day + individual	2

Hour of day and individual were included as random effects in each model. Models 1–4 represent the variables included in the best fitting models (ΔAICc < 2). HK, Hong Kong; SA, South Africa.

**Figure 2:**
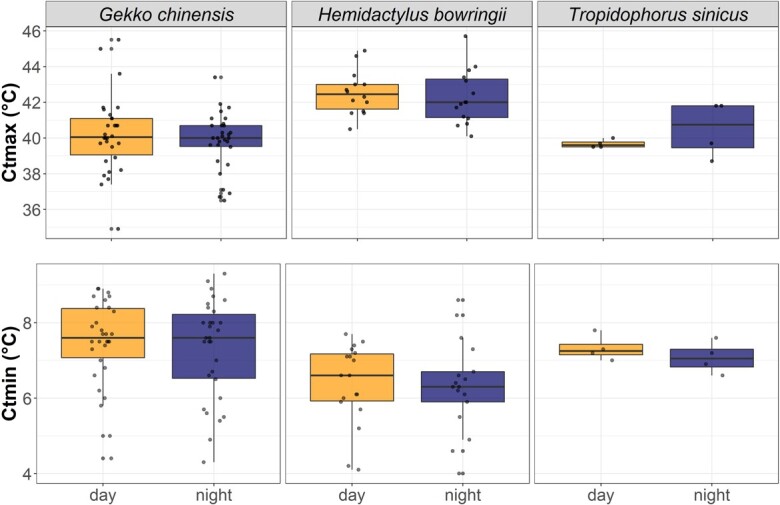
Variation in Ctmax (top) and Ctmin (bottom) between day (orange) and night (dark blue) measurements for three Hong Kong species: two nocturnal geckos (*G. chinensis* and *H. bowringii*) and one cathemeral skink (*T. sinicus*).

In Hong Kong, we detected no significant interaction between variables. Only SVL had an impact on Ctmax (*P < 0.001*), with larger individuals of the same species reaching lower Ctmax (0.11°C lower by millimetre of difference in SVL) (Table 3). This pattern did not change with period (Fig. 2), species nor sex. In South Africa, males achieved a higher Ctmax relative to females (with a difference of 0.93°C on average*, P = 0.01*). None of the variables tested had any significant effect on Ctmin in Hong Kong (*P > 0.25*).

**Table 3:** Statistical outputs of the full averages of best fitting models for the two studied regions

Parameter	Estimate	Std. error	Adjusted SE	*z*-value	Pr (>|t|)
	Model 1: Ctmax – HK ~				
Intercept	47.29	1.49	1.50	31.38	< 2e-16
Period night	−0.74	1.28	1.28	0.580	0.56
SVL	−0.11	0.02	0.02	4.56	< 0.001
Sex, male	−0.17	0.35	0.36	0.466	0.64
Period night^*^sex M	0.33	0.51	0.41	0.647	0.52
Period night^*^SVL	0.007	0.02	0.02	0.394	0.69
	Model 2: Ctmax – SA ~				
Intercept	44.97	0.27	0.27	164.70	< 2e-16
Period night	0.09	0.24	0.24	0.37	0.71
Sex, male	0.93	0.37	0.38	2.46	0.014
	Model 3: Ctmin – HK ~				
Intercept	6.03	1.69	1.70	3.55	< 0.001
SVL	0.02	0.02	0.02	0.78	0.44
Sex, male	0.13	0.25	0.25	0.52	0.61
Species *H. bowringii*	−0.70	0.62	0.62	1.42	0.25
Species *T. sinicus*	−0.05	0.46	0.47	0.11	0.91
	Model 4: Ctmin – SA ~				
Intercept	−0.13	0.30	0.31	0.43	0.24
Period night	1.10	0.56	0.57	1.94	0.05
Sex, male	−0.09	0.25	0.28	0.34	0.73
Species *L. capensis*	2.11	0.57	0.58	3.69	<0.001
Species *T. homalocephala*	1.14	0.66	0.67	1.70	0.09
Period night ^*^ Species *L. capensis*	−2.54	0.71	0.72	3.51	<0.0001
Period night ^*^ Species *T. homalocephala*	−0.03262	0.88	0.90	0.036	0.97
Trial number (2)	−0.23	0.42	0.42	0.54	0.59

**Figure 3:**
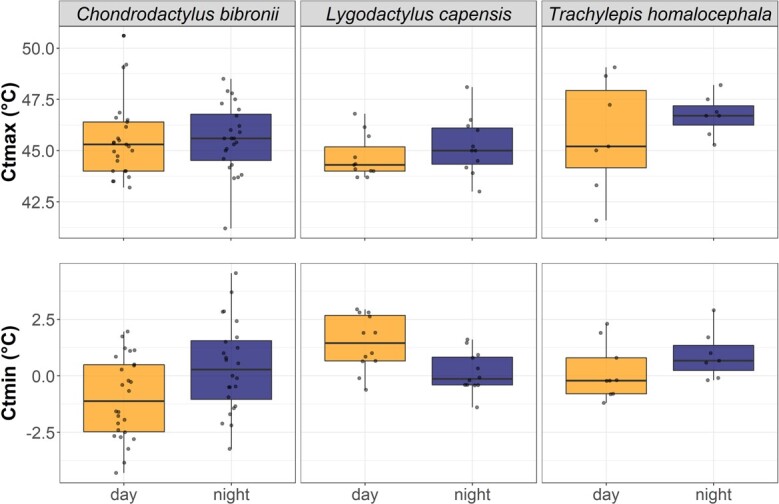
Variation in Ctmin (top) and Ctmax (bottom) between day (orange) and night (dark blue) measurements for three South African species: one nocturnal gecko (*C. bibronii*), one diurnal gecko (*L. capensis*) and one diurnal skink (*T. homalocephala*).

In South Africa, we detected a significant interaction term between period and species and yielded lower Ctmin at night time for *L. capensis* in South Africa (by 2.54°C, *P < 0.001*) while there were no diel differences for the other two species. The main effect ‘period of measurement’ was slightly significant, with lower Ctmin recorded during the night (1.10°C, *P = 0.05*) (Fig. 3). The main effect ‘species’ was significant, with the nocturnal gecko *C. bibronii* having a lower Ctmin (i.e. higher tolerance to cold temperatures) compared to the diurnal gecko *L. capensis* (by 2.13°C, *P* < 0.001) and the diurnal skink *T. homalocephala* (by 1.17°C, *P* = 0.08). Trial number was not significant for Ctmin when included in the main analysis (*P = 0.16*). TTB was only affected by SVL (*P < 0.0001*) in Hong Kong, but period was not an influencing factor neither in Hong Kong nor in South Africa (Supplementary Material Table S1).

## Discussion

While some studies have found differences in thermal tolerance between diurnal and nocturnal species (Arenas-Moreno *et al.*, 2018; Garcia-Robledo *et al.*, 2018; Gaston, 2019), how daily thermal variation might structure ectotherm physiology remains understudied. Our results here, spanning multiple diurnal and nocturnal lizard species, suggest that the timing of measurements (day vs. night) do not consistently or systematically affect estimates of thermal tolerance. There are good reasons to expect differences (e.g. circadian rhythms, variable external/climatic environments) and for other systems, day vs. night thermal tolerance may indeed vary consistently within species and individuals. While we did not find consistent variation in thermal limits with time of measurement, we did find some cases of notable effects, e.g. lower Ctmin at night (as hypothesized) for *L. capensis* in South Africa. Given these results altogether, formal (e.g. adding time of measurement as a covariate or restricting measurement to a single time period) or informal (e.g. acknowledging diel variation in thermal tolerance) incorporation of measurement time should be implemented in thermal tolerance studies. These findings have important consequences for experimental designs, for modelling studies looking to understand geographic and temporal limitations of species distributions, as well as conservation practices examining assisted translocations or invasive species management.

For the lizards in Hong Kong, period (day vs. night) did not affect Ctmin nor Ctmax. However, we did observe that body size was an important factor for Ctmax in Hong Kong, with larger individuals of the same species exhibiting lower tolerance for high temperatures (i.e. narrower thermal tolerance). Such findings have previously been attributed to the mechanistic role of oxygen limitation (Shea *et al.*, 2016), but also to thermal inertia itself, as more time is needed to reach higher body temperature in larger lizards (Porter and Gates, 1969; Stevenson, 1985; Claunch *et al.*, 2021). Bigger animals may also be more subjected to higher heterothermy of the body, and a difference between cloacal (where the measurement is taken) and brain (where loss of righting response is initiated) temperature is more likely to occur (Claunch *et al.*, 2021). Rubalcaba and Olalla-Tárraga (2020) predicted that at lower latitudes, smaller terrestrial lizards were better equipped to resist overheating (i.e. were less vulnerable) than larger species, consistent with our own findings.

Based on the CVH, we expected to find broader thermal tolerance ranges in species that experience a more thermally variable environment and, conversely, relatively little variability in thermal tolerance in species living in more stable environments (Addo-Bediako *et al.*, 2000; Baudier *et al.*, 2018; Gaston, 2009; Ghalambor *et al.*, 2006; Janzen, 1967). In Hong Kong our study species occupy relatively stable thermal environments, as they are mostly nocturnal (*G. chinensis* and *H. bowringii*), or in the case of the cathemeral skink *T. sinicus*, inhabiting streams with a relatively buffered thermal environment (e.g. Polato *et al.*, 2018), which would explain the lack of diel variation. For the lizards in South Africa, period was an important factor for Ctmin, but not Ctmax, in addition to body size. In fact, period of measurement had no effect on Ctmax for any of the species studied here (in both Hong Kong and South Africa), which is consistent with Ctmax being more genetically constrained and evolutionary static than Ctmin (Spicer and Gaston, 1999; Sunday *et al.* 2011; Araújo *et al.*, 2013; Muñoz *et al.*, 2014) and our results do not support bias from captivity time on Ctmax. Our study species in South Africa differed greatly in body size (e.g. *C. bibronii* is about twice the SVL of *L. capensis* and 22 times its body mass), which could partly explain the difference of Ctmin we observed between these two species. Additionally, microhabitat use and temporal niche also vary between species and these ecological differences offer options for reduced exposure to climate extremes (Scheffers *et al.*, 2014). Therefore, following the CVH, we expect the variability of the thermal environment to be buffered by use of microhabitats and variable temporal niches. We observed that the nocturnal gecko *C. bibronii* and diurnal skink *T. homalocephala*, which are primarily rupicolous, show no difference in thermal tolerance ranges between them and no diel variation.

The smallest of the study species, the cape dwarf gecko (*L. capensis*) had lower Ctmin at night, ~2°C lower than during the day. Variable patterns in preferred or selected temperatures in day versus night could result in the difference observed here. For example, Arenas-Moreno *et al.* (2018) found higher preferred/selected temperatures in some lizard species in Mexico during the photophase (day) relative to the scotophase (night). Originating from the tropical west of South Africa, *L. capensis* has recently spread and established in the Western Cape, likely introduced via human pathways (ornamental plant industry and tourism) (Rebelo *et al.*, 2019). The native range of this species could explain the reduced tolerance for cold temperatures compared to the two other species (also in line with CVH). These results highlight the complexity in species adaptation to both seasonal and diel variation in temperature in their current distributions (or introduced) (Esch *et al.*, 2017; Ghalambor *et al.*, 2006), as well as the seasonal and diel variation in their historic (or native) ranges, e.g. in the case of niche conservatism (Araújo *et al.*, 2013). For only this species in our study, the timing of measurement influenced thermal tolerance. This finding then demonstrates how such differences in day versus night-time thermal tolerance can be context specific (e.g. influenced by introduction history, climate in native range, or other factors).

We pooled our data into two periods (night vs. day) but there may be specific time points, especially during the second half of the night (i.e. after midnight as in Johnson, 1972), which could trigger lower thermal tolerance for cold temperatures in diurnal species in temperate areas following the CVH. However, our sampling design (comparison of multiple species during the same season in two different geographic areas) as well as logistical constraints did not allow us to investigate this hour-by-hour variation during the entire diel cycle. Similarly, different populations of *C. bibronii* and *G. chinensis* may have had different thermal tolerances as a result of experiencing different thermal environments (Supplementary Material Fig. S3), but we found no additional effect of population on the presence or absence of diel variation in thermal tolerance. Additional sources of variation, outside the scope of our study, may also be important in driving diel variation in thermal tolerance. Phenotypic plasticity is fundamental for organisms in dealing with thermal variation during their lifespan (Basson and Clusella-Trullas, 2015). To account for differences between populations and collection dates, we acclimated all our lizards at the same temperature regime for a period of 7 days before conducting experiments. We found no effect of the length of time in captivity on our measurements of Ctmin and Ctmax. However, individuals were still free to thermoregulate in the limited range offered to them and this could have generated individual responses under the standardized ramping rate. Behavioural thermoregulation is an important factor of the realized thermal niche of ectotherms (Bonebrake *et al.*, 2014; Sunday *et al.*, 2014) and can not only promote evolution but also likely shelters selection on thermal traits (i.e. ‘Bogert effect’; Huey *et al.*, 2003; Muñoz *et al.*, 2014). Therefore, while our study provides important information on diel variation in physiological thermal limits, further investigation on day vs. night tracking of preferred temperature and other physiological traits (metabolism, water loss) would enhance assessment of these species’ vulnerability/exposure to climate change.

Despite our sampling range covering a wide range in terms of species, temporal niches, morphologies, locations and habitats, our results reveal no consistent diel variation in thermal tolerance. Rather, we show that this response may be species-specific. Timing of thermal limit measurements then may be an important consideration in ectotherm climate responses in some cases, but the differences in estimates are unlikely to deviate in clear nor predictable ways.

## Funding

This work was supported by Hong Kong Research Grant Council General Research Fund (17152316 to T.C.B. and S.C.-T.). Equipment usage in South Africa was supported by South African National Research Foundation funding to S.C.-T.

## Data Availability

Data for this study is available from the Dryad Digital Repository: https://doi.org/10.5061/dryad.pc866t1qw.

## Ethics approval

Field sites in South Africa were private and we obtained approval from landowners prior to sampling. We therefore address our deepest gratitude to the Le Roux family, Mr Piketberg, Mr Linde, Mr and Mrs Nieuwouldt and the Cape Garden plant nursery, who kindly agreed to let us sample around their properties. This research was conducted with the approval of the Hong Kong Department of Health (Cap. 340, 18–344 and 18–345) and the University of Hong Kong’s Committee of the Use of Live Animals in Teaching & Research (4675–18). We also obtained approval from Cape Nature (permit CN44-59-5425) and the Research Ethics Committee: Animal Care and Use (protocol no. 7717) from Stellenbosch University, South Africa.

## Supplementary Material

Web_Material_coac020

## References

[ref1] Addo-Bediako A, Chown SL, Gaston KJ (2000) Thermal tolerance, climatic variability and latitude. Proc Biol Sci 267: 739–745. 10819141 10.1098/rspb.2000.1065 PMC1690610

[ref2] Adesina AJ, Kumar KR, Sivakumar V (2016) Aerosol-cloud-precipitation interactions over major cities in South Africa: impact on regional environment and climate change. Aerosol Air Qual Res 16: 195–211.

[ref3] Allen JL, Chown SL, Janion-Scheepers C, Clusella-Trullas S (2016) Interactions between rates of temperature change and acclimation affect latitudinal patterns of warming tolerance. Conserv Physiol 4: cow053. 27933165 10.1093/conphys/cow053 PMC5142048

[ref4] Angilletta MJ (2009) Thermal Adaptation. Oxford University Press, Oxford and New York.

[ref5] Araújo MB, Ferri-Yáñez F, Bozinovic F, Marquet PA, Valladares F, Chown SL (2013) Heat freezes niche evolution. Ecol Lett 16: 1206–1219. 23869696 10.1111/ele.12155

[ref6] Arenas-Moreno DM, Santos-Bibiano R, Muñoz-Nolasco FJ, Charruau P, Méndez-de la Cruz FR (2018) Thermal ecology and activity patterns of six species of tropical night lizards (Squamata: Xantusiidae: Lepidophyma) from Mexico. J Therm Biol 75: 97–105. 30017058 10.1016/j.jtherbio.2018.06.001

[ref7] Bai CM, Ma G, Cai WZ, Ma CS (2019) Independent and combined effects of daytime heat stress and night-time recovery determine thermal performance. Biol Open 8: bio038141. 30837225 10.1242/bio.038141 PMC6451327

[ref8] Bartón K (2020) MuMIn: multi-model inference. R package version 1.43.17. https://cran.r-project.org/package=MuMIn.

[ref9] Basson CH, Clusella-Trullas S (2015) The behavior-physiology nexus: behavioral and physiological compensation are relied on to different extents between seasons. Physiol Biochem Zool 88: 384–394. 26052635 10.1086/682010

[ref10] Bates D, Sarkar D, Bates MD, Matrix L (2007) The lme4 package. R package version 2: 74. https://cran.r-project.org/package=lme4.

[ref11] Baudier KM, D’Amelio CL, Malhotra R, O’Connor MP, O’Donnell S (2018) Extreme insolation: climatic variation shapes the evolution of thermal tolerance at multiple scales. Am Nat 192: 347–359. 30125235 10.1086/698656

[ref12] Beck HE, Zimmermann NE, McVicar TR, Vergopolan N, Berg A, Wood EF (2018) Present and future Köppen-Geiger climate classification maps at 1-km resolution. Sci Data 5: 1–12. 30375988 10.1038/sdata.2018.214 PMC6207062

[ref13] Bennett JM, Calosi P, Clusella-Trullas S, Martínez B, Sunday J, Algar AC, Araújo MB, Hawkins BA, Keith S, Kühn I et al. (2018) GlobTherm, a global database on thermal tolerances for aquatic and terrestrial organisms. Sci Data 5: 1–7. 29533392 10.1038/sdata.2018.22 PMC5848787

[ref14] Bennett JM, Sunday J, Calosi P, Villalobos F, Martínez B, Molina-Venegas R, Araújo MB, Algar AC, Clusella-Trullas S, Hawkins BA et al. (2021) The evolution of critical thermal limits of life on Earth. Nat Commun 12: 1–9. 33608528 10.1038/s41467-021-21263-8 PMC7895938

[ref15] Besson AA, Cree A (2011) Integrating physiology into conservation: an approach to help guide translocations of a rare reptile in a warming environment. Anim Conserv 14: 28–37.

[ref77] Bonebrake TC, Boggs CL, Stamberger JA, Deutsch CA, Ehrlich PR (2014) From global change to a butterfly flapping: biophysics and behaviour affect tropical climate change impacts. Proc Royal Soc B 281: 20141264. 10.1098/rspb.2014.1264 PMC4173678 25165769

[ref16] Bonino MF, Azócar DLM, Schulte JA II, Abdala CS, Cruz FB (2015) Thermal sensitivity of cold climate lizards and the importance of distributional ranges. Fortschr Zool 118: 281–290. 10.1016/j.zool.2015.03.001 26066005

[ref17] Brattstrom BH (1970) Thermal acclimation in Australian amphibians. Comp Biochem Physiol 35: 69–103. 10.1016/0010-406x(68)90961-4 5689525

[ref18] Buckley LB, Ehrenberger JC, Angilletta MJ (2015) Thermoregulatory behaviour limits local adaptation of thermal niches and confers sensitivity to climate change. Funct Ecol 29: 1038–1047.

[ref19] Burnham KP, Anderson DR (2004) Multimodel inference: understanding AIC and BIC in model selection. Sociol Methods Res 33: 261–304.

[ref20] Chong G, Heatwole H, Firth BT (1973) Panting thresholds of lizards—II. Diel variation in the panting threshold of *Amphibolurus muricatus*. Comp Biochem Physiol 46: 827–829. 10.1016/0300-9629(73)90131-x 4148170

[ref21] Claunch NM, Nix E, Royal AE, Burgos LP, Corn M, DuBois PM, Ivey KN, King EC, Rucker KA, Shea TK et al. (2021) Body size impacts critical thermal maximum measurements in lizards. J Exp Zool 335: 96–107. 10.1002/jez.2410 32851814

[ref22] Clusella-Trullas S, Blackburn TM, Chown SL (2011) Climatic predictors of temperature performance curve parameters in ectotherms imply complex responses to climate change. Am Nat 177: 738–751. 21597251 10.1086/660021

[ref23] Clusella-Trullas S, Chown SL (2014) Lizard thermal trait variation at multiple scales: a review. J Comp Physiol B 184: 5–21. 23989339 10.1007/s00360-013-0776-x

[ref24] Cooke SJ, Hinch SG, Donaldson MR, Clark TD, Eliason EJ, Crossin GT, Farrell AP (2012) Conservation physiology in practice: how physiological knowledge has improved our ability to sustainably manage Pacific salmon during up-river migration. Philos Trans R Soc Lond B Biol Sci 367: 1757–1769. 22566681 10.1098/rstb.2012.0022 PMC3350662

[ref25] Cooke SJ, Kassler TW, Philipp DP (2001) Physiological performance of largemouth bass related to local adaptation and interstock hybridization: implications for conservation and management. J Fish Biol 59: 248–268.

[ref26] Cree A, Hare KM, Nelson NJ, Chukwuka C, Virens J (2020) How thermal ecophysiology assists the conservation of reptiles: case studies from New Zealand’s endemic fauna. In Conservation Physiology: Applications for Wildlife Conservation and Management. Oxford University Press, pp. 269–286

[ref27] Davy R, Esau I, Chernokulsky A, Outten S, Zilitinkevich S (2017) Diurnal asymmetry to the observed global warming. Int J Climatol 37: 79–93.

[ref78] Deutsch CA, Tewksbury JJ, Huey RB, Sheldon KS, Ghalambor CK, Haak DC, Martin, PR (2008) Impacts of climate warming on terrestrial ectotherms across latitude. Proc Natl Acad Sci 105: 6668–6672. 18458348 10.1073/pnas.0709472105 PMC2373333

[ref28] Esch C, Jimenez JP, Peretz C, Uno H, O’Donnell S (2017) Thermal tolerances differ between diurnal and nocturnal foragers in the ant *Ectatomma ruidum*. Insect Soc 64: 439–444.

[ref29] Garcia-Robledo C, Chuquillanqui H, Kuprewicz EK, Escobar-Sarria F (2018) Lower thermal tolerance in nocturnal than in diurnal ants: a challenge for nocturnal ectotherms facing global warming. Ecol Entomol 43: 162–167.

[ref30] Gaston KJ (2009) Geographic range limits: achieving synthesis. Proc Biol Sci 276: 1395–1406. 19324809 10.1098/rspb.2008.1480 PMC2677218

[ref31] Gaston KJ (2019) Nighttime ecology: the “nocturnal problem” revisited. Am Nat 193: 481–502. 30912975 10.1086/702250

[ref32] Ghalambor CK, Huey RB, Martin PR, Tewksbury JJ, Wang G (2006) Are mountain passes higher in the tropics? Janzen's hypothesis revisited. Integr Comp Biol 46: 5–17. 21672718 10.1093/icb/icj003

[ref33] Grigg JW, Buckley LB (2013) Conservatism of lizard thermal tolerances and body temperatures across evolutionary history and geography. Biol Lett 9: 20121056. 23325735 10.1098/rsbl.2012.1056 PMC3639756

[ref34] Grueber CE, Nakagawa S, Laws RJ, Jamieson IG (2011) Multimodel inference in ecology and evolution: challenges and solutions. J Evol Biol 24: 699–711. 21272107 10.1111/j.1420-9101.2010.02210.x

[ref35] Gvoždík L (2018) Just what is the thermal niche? Oikos 127: 1701–1710.

[ref36] Huey RB, Hertz PE, Sinervo B (2003) Behavioral drive versus behavioral inertia in evolution: a null model approach. Am Nat 161: 357–366. 12699218 10.1086/346135

[ref37] Huey RB, Kingsolver JG (1989) Evolution of thermal sensitivity of ectotherm performance. Trends Ecol Evol 4: 131–135. 21227334 10.1016/0169-5347(89)90211-5

[ref38] Hutchison VH (1961) Critical thermal maxima in salamanders. Physiol Zool 34: 92–125.

[ref39] Jacobson ER, Whitford WG (1970) The effect of acclimation on physiological responses to temperature in the snakes, *Thamnophis proximus* and *Natrix rhombifera*. Comp Biochem Physiol 35: 439–449.

[ref40] Janzen DH (1967) Why mountain passes are higher in the tropics. Am Nat 101: 233–249.

[ref41] Johnson CR (1972) Thermal relations and daily variation in the thermal tolerance in *Bufo marinus*. J Herpetol 6: 35–38.

[ref79] Kearney MR, Isaac AP, Porter WP (2014) microclim: Global estimates of hourly microclimate based on long-term monthly climate averages. Sci Data 1: 1–9. 10.1038/sdata.2014.6 PMC4387738 25977764

[ref42] Kern P, Cramp RL, Franklin CE (2015) Physiological responses of ectotherms to daily temperature variation. J Exp Biol 218: 3068–3076. 26254318 10.1242/jeb.123166

[ref43] Kubisch EL, Corbalán V, Ibargüengoytía NR, Sinervo B (2016) Local extinction risk of three species of lizard from Patagonia as a result of global warming. Can J Zool 94: 49–59.

[ref44] Leal M, Gunderson AR (2012) Rapid change in the thermal tolerance of a tropical lizard. Am Nat 180: 815–822. 23149405 10.1086/668077

[ref45] Lindenmayer D, Scheele B (2017) Do not publish. Science 356: 800–801. 28546170 10.1126/science.aan1362

[ref46] Liwanag HE, Haro D, Callejas B, Labib G, Pauly GB (2018) Thermal tolerance varies with age and sex for the nonnative Italian Wall lizard (*Podarcis siculus*) in Southern California. J Therm Biol 78: 263–269. 30509645 10.1016/j.jtherbio.2018.10.010

[ref47] Llewelyn J, Macdonald SL, Moritz C, Martins F, Hatcher A, Phillips BL (2018) Adjusting to climate: acclimation, adaptation and developmental plasticity in physiological traits of a tropical rainforest lizard. Integr Zool 13: 411–427. 29316349 10.1111/1749-4877.12309

[ref48] Lowe CH, Vance VJ (1955) Acclimation of the critical thermal maximum of the reptile *Urosaurus ornatus*. Science 122: 73–74. 17748800 10.1126/science.122.3158.73

[ref49] Madliger CL, Love OP, Hultine KR, Cooke SJ (2018) The conservation physiology toolbox: status and opportunities. Conserv Physiol 6: coy029. 29942517 10.1093/conphys/coy029 PMC6007632

[ref50] Muñoz MM, Stimola MA, Algar AC, Conover A, Rodriguez AJ, Landestoy MA, Bakken GS, Losos JB (2014) Evolutionary stasis and lability in thermal physiology in a group of tropical lizards. Proc Biol Sci 281: 20132433. 24430845 10.1098/rspb.2013.2433 PMC3906933

[ref51] Nakagawa S, Schielzeth H (2013) A general and simple method for obtaining R2 from generalized linear mixed-effects models. Methods Ecol Evol 4: 133–142.

[ref52] Pincebourde S, Suppo C (2016) The vulnerability of tropical ectotherms to warming is modulated by the microclimatic heterogeneity. Integr Comp Biol 56: 85–97. 27371561 10.1093/icb/icw014

[ref53] Polato NR, Gill BA, Shah AA, Gray MM, Casner KL, Barthelet A, Messer PW, Simmons MP, Guayasamin JM, Encalada AC et al. (2018) Narrow thermal tolerance and low dispersal drive higher speciation in tropical mountains. Proc Natl Acad Sci U S A 115: 12471–12476. 30397141 10.1073/pnas.1809326115 PMC6298121

[ref54] Pontes-da-Silva E, Magnusson WE, Sinervo B, Caetano GH, Miles DB, Colli GR, Lm D-V, Fenker J, Santos JC, Werneck FP (2018) Extinction risks forced by climatic change and intraspecific variation in the thermal physiology of a tropical lizard. J Therm Biol 73: 50–60. 29549991 10.1016/j.jtherbio.2018.01.013

[ref80] Porter WP, Gates DM (1969) Thermodynamic equilibria of animals with environment. Ecol Monogr 39: 227–244.

[ref55] Rebelo AD, Bates MF, Burger M, Branch WR, Conradie W (2019) Range expansion of the common dwarf gecko, *Lygodactylus capensis*: South Africa’s most successful reptile invader. Herpet Notes 12: 643–650.

[ref56] Ribeiro PL, Camacho A, Navas CA (2012) Considerations for assessing maximum critical temperatures in small ectothermic animals: insights from leaf-cutting ants. PLoS One 7: e32083. 22384147 10.1371/journal.pone.0032083 PMC3286443

[ref57] RStudio Team (2019) RStudio: Integrated Development for R. RStudio, Inc., Boston, MA. http://www.rstudio.com/.

[ref58] Rubalcaba JG, Olalla-Tárraga MÁ (2020) The biogeography of thermal risk for terrestrial ectotherms: scaling of thermal tolerance with body size and latitude. J Anim Ecol 89: 1277–1285. 31990044 10.1111/1365-2656.13181

[ref59] Scheffers BR, Edwards DP, Diesmos A, Williams SE, Evans TA (2014) Microhabitats reduce animal's exposure to climate extremes. Glob Change Biol 20: 495–503. 10.1111/gcb.12439 24132984

[ref60] Sharma NK, Akhtar MS, Pandey N, Singh R, Singh AK (2015) Seasonal variation in thermal tolerance, oxygen consumption, antioxidative enzymes and non-specific immune indices of Indian hill trout, *Barilius bendelisis* (Hamilton, 1807) from central Himalaya, India. J Therm Biol 52: 166–176. 26267511 10.1016/j.jtherbio.2015.07.005

[ref61] Shea TK, DuBois PM, Claunch NM, Murphey NE, Rucker KA, Brewster RA, Taylor EN (2016) Oxygen concentration affects upper thermal tolerance in a terrestrial vertebrate. Comp Biochem Physiol 199: 87–94. 10.1016/j.cbpa.2016.05.026 27264957

[ref62] Sinervo B, Mendez de la Cruz FR, Miles DB, Heulin B, Bastiaans E, Villagrán-Santa Cruz M, Lara-Resendiz R, Martínez-Méndez M, Calderón-Espinosa ML, Meza-Lázaro RN et al. (2010) Erosion of lizard diversity by climate change and altered thermal niches. Science 328: 894–899. 20466932 10.1126/science.1184695

[ref63] Spellerberg IF (1973) Critical minimum temperatures of reptiles. In Effects of Temperature on Ectothermic Organisms. Springer, Berlin, Heidelberg, pp. 239–247.

[ref81] Spicer J, Gaston K (1999) Physiological diversity: ecological implications. John Wiley & Sons.

[ref64] Stevenson RD (1985) The relative importance of behavioral and physiological adjustments controlling body temperature in terrestrial ectotherms. Am Nat 126: 362–386.

[ref65] Stocker TF, Qin D, Plattner G-K, Tignor M, Allen SK, Boschung J, Nauels A, Xia Y, Bex V, Midgley PM (2013) Summary for Policymakers. In Climate Change 2013: The Physical Science Basis Contribution of Working Group I to the Fifth Assessment Report of the Intergovernmental Panel on Climate Change. Cambridge University Press, Cambridge, United Kingdom and New York, USA

[ref66] Sunday JM, Bates AE, Dulvy NK (2011) Global analysis of thermal tolerance and latitude in ectotherms. Proc Biol Sci 278: 1823–1830. 21106582 10.1098/rspb.2010.1295 PMC3097822

[ref67] Sunday JM, Bates AE, Kearney MR, Colwell RK, Dulvy NK, Longino JT, Huey RB (2014) Thermal-safety margins and the necessity of thermoregulatory behavior across latitude and elevation. Proc Natl Acad Sc U S A 111: 5610–5615. 24616528 10.1073/pnas.1316145111 PMC3992687

[ref68] Taylor EN, Diele-Viegas LM, Gangloff EJ, Hall JM, Halpern B, Massey MD, Rödder D, Rollinson N, Spears S, Sun B et al. (2021) The thermal ecology and physiology of reptiles and amphibians: a user's guide. J Exp Zool Part B Mol Dev Evol 335: 13–44. 10.1002/jez.2396 32638552

[ref69] Terblanche JS, Hoffmann AA, Mitchell KA, Rako L, le Roux PC, Chown SL (2011) Ecologically relevant measures of tolerance to potentially lethal temperatures. J Exp Biol 214: 3713–3725. 22031735 10.1242/jeb.061283

[ref70] Turko AJ, Leclair AT, Mandrak NE, Drake DAR, Scott GR, Pitcher TE (2021) Choosing source populations for conservation reintroductions: lessons from variation in thermal tolerance among populations of the imperilled redside dace. J Fish Res Board Can 78: 1347–1355.

[ref71] Virens J, Cree A (2019) Pregnancy reduces critical thermal maximum, but not voluntary thermal maximum, in a viviparous skink. J Comp Physiol B 189: 611–621. 31493184 10.1007/s00360-019-01230-y

[ref72] Ward R, Blandon IR, King TL, Beitinger TL (1993) Comparisons of critical thermal maxima and minima of juvenile red drum (*Sciaenops ocellatus*) from Texas and North Carolina. NEGS 13: 23–28.

[ref73] Weldon CW, Terblanche JS, Chown SL (2011) Time-course for attainment and reversal of acclimation to constant temperature in two Ceratitis species. J Therm Biol 36: 479–485.

[ref74] Willhite C, Cupp PV Jr (1982) Daily rhythms of thermal tolerance in *Rana clamitans* (Anura: Ranidae) tadpoles. Comp Biochem Physiol A 72: 255–257.

[ref75] Xu XF, Ji X (2006) Ontogenetic shifts in thermal tolerance, selected body temperature and thermal dependence of food assimilation and locomotor performance in a lacertid lizard, Eremias brenchleyi. Comp Biochem Physiol A Mol Integr Physiol 143: 118–124. 16380280 10.1016/j.cbpa.2005.11.004

[ref76] Zerebecki RA, Sorte CJ (2011) Temperature tolerance and stress proteins as mechanisms of invasive species success. PLoS One 6: e14806. 21541309 10.1371/journal.pone.0014806 PMC3082523

