# Supplementary material for: No consistent effect of daytime versus night-time measurement of thermal tolerance in nocturnal and diurnal lizards
Source: Conserv Physiol. 2022 Apr 20;10(1):coac020. doi: 10.1093/conphys/coac020 (PMC9040285; doi:10.1093/conphys/coac020)
Supplement: Web_Material_coac020 [file web_material_coac020.docx]

## **Supplemental**


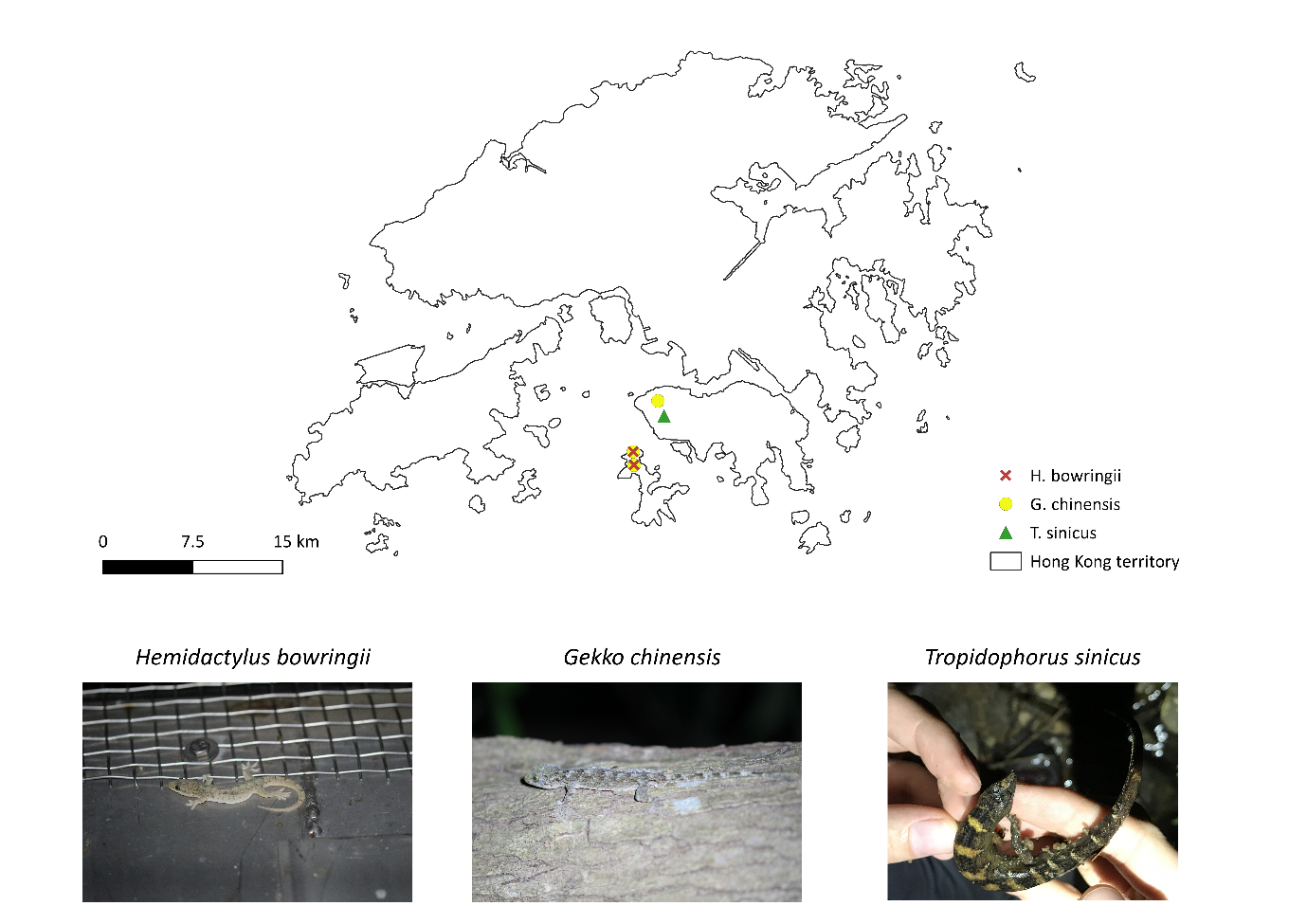


Figure S1. Sampling sites and species of interest in Hong Kong SAR, China.


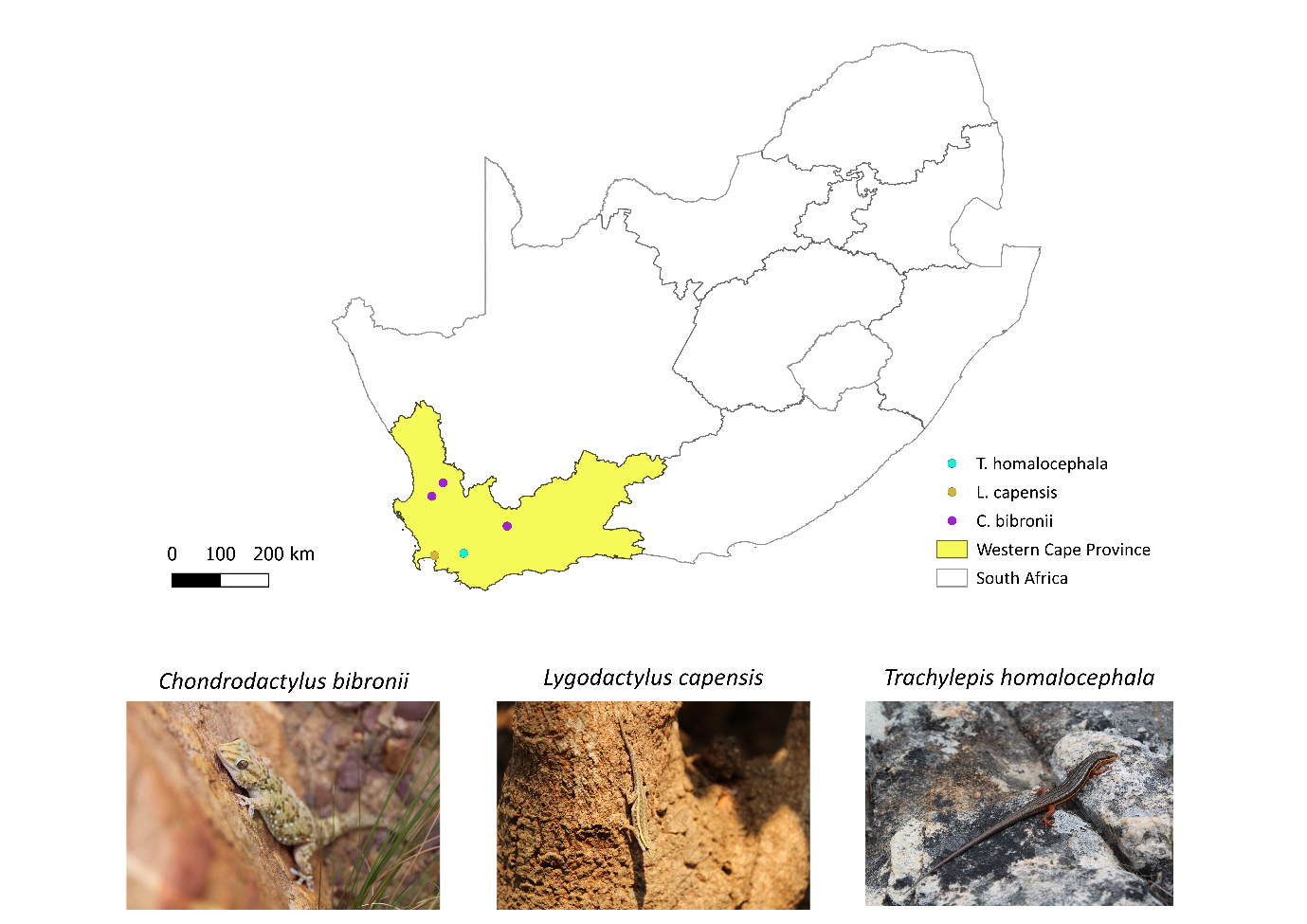


Figure S2. Sampling sites and species of interest in the Western Cape, South Africa.


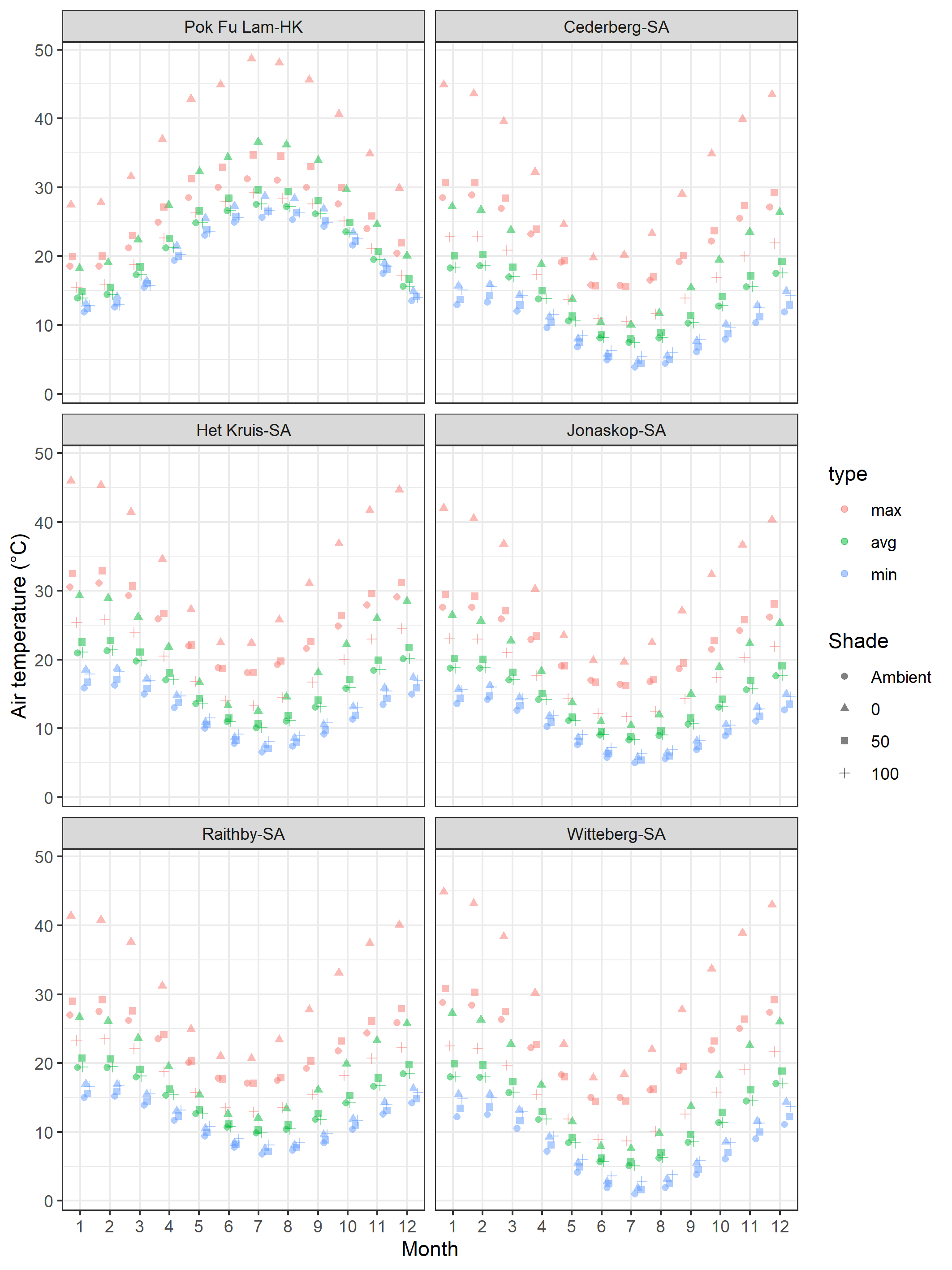


Figure S3. Temperatures at five study sites in South Africa (“SA”) and one site in Hong Kong (“HK”). Temperature profiles include ambient temperature at 120cm from the ground (and at 50% shade) and rock substrate temperatures at three levels of shade (0%, 50%, and 100%). Coloured points indicate maximum (red), average (green), and minimum (blue) daily temperatures for the middle day of each month, as presented in the “microclim” dataset (Kearney et al. 2014). Among South African species, *C. bibronii* was present in Cederberg, Het Kruis, and Witteberg. *T. homalocephala* in Jonaskop, *L. capensis* in Raithby.

Table S1. Statistical outputs of the full averages of best fitting models for the two studied regions for thermal tolerance breath (TTB), calculated as the difference between Ctmax and Ctmin.

| **Parameter** | **Estimate** | **Std. error** | **Adjusted SE** | **z value** | **Pr (>\|t\|)** |
| --- | --- | --- | --- | --- | --- |
|  | Model 1: TTB – HK ~ | | | | |
| Intercept | 43.07 | 2.84 | 2.86 | 15.05 | < 2e-16 |
| Period Night | -0.54 | 1.31 | 1.32 | 0.41 | 0.69 |
| SVL | -0.17 | 0.05 | 0.05 | 3.51 | < 0.0001 |
| Sex Male | -0.05 | 0.27 | 0.28 | 0.19 | 0.85 |
| Species *H. bowringii* | 0.30 | 0.77 | 0.77 | 0.39 | 0.70 |
| Species *T. sinicus* | -0.02 | 0.52 | 0.53 | 0.05 | 0.96 |
| Period Night * SVL | 0.007 | 0.02 | 0.02 | 0.34 | 0.73 |
|  | Model 2: TTB – SA ~ | | | | |
| Intercept | 45.57 | 0.83 | 0.84 | 54.45 | < 2e-16 |
| Period Night | -0.75 | 0.82 | 0.82 | 0.91 | 0.37 |
| Sex Male | 0.78 | 0.77 | 0.78 | 1.00 | 0.32 |
| Species *L. capensis* | -1.49 | 1.60 | 1.61 | 0.93 | 0.35 |
| Species *T. homalocephala* | -0.52 | 0.90 | 0.91 | 0.58 | 0.56 |
| Period Night * Species *L. capensis* | 1.70 | 1.84 | 1.85 | 0.92 | 0.36 |
| Period Night * Species *T. homalocephala* | 0.68 | 1.19 | 1.20 | 0.57 | 0.57 |
